# Dr. Waldon, on Scarlatina

**Published:** 1806-12

**Authors:** John Waldon

**Affiliations:** Bodmin, Cornwall


### *To the Editors of the Medical and Physical Journal.*

GENTLEMEN,

IN the autumn of 1805, the Scarlatina Anginosa made its appearance in the north of Devon, under the most severe form; and in a great and alarming proportion of cases, resisted the usual treatment recommended by authors, though the most approved remedies were early administered, and in such doses as appeared to insure success. The disease most commonly commenced with cold shiverings, great prostration of strength, dejection of spirits, perpetual anxiety, and oppression of the præcordia; together with dull and watery eyes.

The stomach was frequently affected with sickness and vomiting, and not unfrequently the intestines were in too lax a state. The pulse were also quick, weak, unequal, and fluttering; and in the space of eight or ten hours, an efflorescence made its appearance on the skin. The patients scarcely ever complained of much pain in the throat, or difficulty in swallowing, but a distressing dryness almost always occurred; and on inspecting the internal fauces, the mucous membrane had assumed a purple hue with beginning apthæ; which, however, soon became of a dark mahogany colour, and in the space of four days and sometimes sooner, from the first attack, several patients were cut off. The disease was also so very contagious, that almost every member of some families, old and young, in one parish, were taken when it first appeared, and before the atmosphere of the infected houses had been oxygenized by the nitrous or muriatic acids; so that in some families, two or more were lying dead at the same time. Emetics, even on the first attack, were not of service, but on the contrary, seemed to exhaust the patient, and to increase the depression of strength. The cinchona was also early early administered in large doses, and at short intervals, together with a liberal use of port wine and the mineral acids; stimulating gargarisms; blisters, &c. without the least advantage whatever. In this state of alarm and anxiety, I was willing to ascertain the antiseptic and tonic powers of the carbonic acid gas; and I began by ordering a scruple of the kali preparatum in an ounce of the mistura camphorata, washing down instantly after with half an ounce of the succus limonum, and an equal quantity of the spiritus cinnamomi, so that the evolution of the gas should wholly take place in the fauces, œsophagus, and stomach. The kali and acid were also ordered to be repeated every hour or two, according to the urgency of circumstances; and I had the extreme satisfaction soon to witness its decidedly good effects, as every bad symptom was immediately arrested, and not a patient died after I commenced this mode of treatment. When the neutral salts formed by the kali and acid, occasioned loose stools, or aggravated the purgings, as was sometimes the case, I ordered a few drops of the tinctura opii with the gum kino, to be added to the draughts, which immediately corrected them. Before quitting the subject of this very useful and important medicine, I beg leave also to recommend it in every case of typhus, more particularly where petechiæ, and other symptoms of putrefaction of the fluids have manifested themselves; in short, in all cases where the vis vitæ is greatly reduced: as I have for many years been in the habit of administering it under such circumstances, with the most signal success.

As the prepared kali is an extremely nauseous medicine, I suggested the idea of getting it super-saturated with carbonic acid, which gives it a chrystallized form, without deliquescing in the air, adds considerably to its efficacy, and at the same time destroys its unpleasant taste. This preparation of the kali also being so very loosely combined with the excess of carbonic acid, as to be easily decomposed in the stomach, I have given it with great success in many stomach and calculous complaints, instead of the aqua mephitica alkalina. The super carbonated kali is very accurately prepared by a Mr. Hill, an ingenious chymist of Exeter.

I am, &c.

*Bodmin, Cornwall,*

*Nov.* 5, 1806.

JOHN WALDON, M.D.

